# Synthesis of Lignin-Based MMA-*co*-BA Hybrid Resins from Cornstalk Residue via RAFT Miniemulsion Polymerization and Their Characteristics

**DOI:** 10.3390/polym13060968

**Published:** 2021-03-22

**Authors:** Yuzhi Xu, Ning Li, Guangbin Wang, Chunpeng Wang, Fuxiang Chu

**Affiliations:** 1 Institute of Chemical Industry of Forest Products, Chinese Academy of Forestry, Nanjing 210042, China; lining20140214@163.com (N.L.); wanggb_robin@163.com (G.W.); 2 Research Institute of Forestry New Technology, Chinese Academy of Forestry, Beijing 100091, China; 3 Co-Innovation Center of Efficient Processing and Utilization of Forest Resources, Jiangsu Province, Nanjing Forestry University, Nanjing 210037, China

**Keywords:** lignin, macromolecular chain transfer agent, RAFT miniemulsion polymerization, thermal behavior, mechanical performance

## Abstract

The conversion of cornstalk lignin derived from the co-product of bio-refinery into value-added products such as polymeric material has remarkable environmental and economic potential. A novel bio-based methyl methacrylate copolymerized with butyl acrylate (MMA-*co*-BA) hybrid resin in our research was prepared by the reversible addition–fragmentation chain transfer method using lignin-graft-polyacrylamide (lignin-g-PAM) as a bio-derived macromolecular chain transfer agent. The molecular architecture of lignin-g-PAM and the lignin-based MMA-*co*-BA hybrid resin was elucidated using ^1^H nuclear magnetic resonance and attenuated total reflectance–Fourier transform infrared. The thermal behavior and mechanical performance of the resultant lignin-based MMA-*co*-BA hybrid resins were also investigated through thermogravimetric analysis, differential scanning calorimetry, and a stress–strain test, respectively. The lignin-based acrylate resins system exhibited structure-related thermal and mechanical properties. Compared with pure MMA-*co*-BA resin, the incorporation of lignin into various lignin-based MMA-*co*-BA graft copolymers resulted in an improved tensile strength and a higher Young’s modulus. This research could provide not only a new avenue to utilize waste biomass for high-value applications, but also a reference for designing new materials for coatings or adhesives.

## 1. Introduction

Acrylate resins, which are versatile copolymers produced basically from petroleum resources, play an important role in elastomer, coating, adhesive, and composite industries [1,2,3,4]. In the context of rising global warming and limited fossil fuel reserves, researchers in related fields have been making the utmost effort to search for regenerative resources for the preparation of acrylic resins with sustainability, a lower carbon footprint, and a relatively low cost [5,6,7]. Therefore, many reports have previously been published on the preparation of environmentally friendly acrylic polymers from a wide variety of sustainable resources [8], including terpene [9,10,11], tannins [12], lactone [13], lactic acid [14,15], chitosan [16], vegetable oils [17,18,19,20], cardanol [21,22,23], and lignin [24,25]. 

Lignin, industrially generated as a byproduct of paper and bio-refinery, is the second most abundant biomass on the Earth. Therefore, utilizing this sustainable resource can be cost-effective and can lead to much higher profits for these two industries, as well as producing a higher environmental value [26,27,28]. The active functional groups in lignin, such as methoxyl, carboxyl, and hydroxyl, offer a number of opportunities for synthesis of lignin-based green materials [29,30,31]. Lignin from cornstalk has been investigated for a number of years [32,33,34,35], and reports focus mainly on structure characterization of lignin [32,33], preparation of lignin-based functional materials [34], and composites based on modified lignin [35]. In addition, acrylamide can react with itself and other various monomers [36]. These homopolymers and copolymers have been found to have numerous applications [37], especially in the paper industry, in wastewater treatment, and as soil conditioners to improve soil texture.

In our previous study, the preparation and properties of lignin-grafted acrylic copolymers from lignocellulosic butanol residue via free radical polymerization were elucidated in some detail [24]. Some researchers have focused on the synthesis of renewable acrylic resins using controlled radical polymerization [38], atom transfer radical polymerization (ATRP) [39], and reversible addition–fragmentation transfer (RAFT) reactions [40].

As compared to conventional free radical methods, living radical polymerization can confer numerous benefits [41,42,43]. These involve the capability of controlling molecular weight and polydispersity and of synthesizing block copolymers, as well as materials of complex polymeric architectures not readily prepared using other techniques [44,45,46,47]. What distinguishes the RAFT process from other methods of living radical polymerization is that it can be carried out under various reaction conditions for a wide range of monomers [48,49,50,51]. The RAFT approach has been successfully used in the polymerization of acrylic monomers [52,53,54,55].

A survey of the literature shows that most of the bio-based acrylate resins to date have been designed and synthesized using free radical polymerization [24,56,57], ATRP [25,58], and RAFT polymerization [40] in organic media. To the best of our knowledge, no research has been reported on the preparation of (meth) acrylate resins utilizing a lignin-based copolymer as a macromolecular chain transfer agent by RAFT miniemulsion polymerization.

The chief goal of this study was to prepare lignin-based hybrid acrylate resins via RAFT miniemulsion polymerization. In this research, lignin xanthate was first synthesized and then reacted with acrylamide to produce a lignin-based polymeric RAFT agent. The chemical functionality of the lignin-graft-polyacrylamide (lignin-g-PAM) was also investigated. The methyl methacrylate copolymerized with butyl acrylate (MMA-*co*-BA) hybrid latex was synthesized using lignin-g-PAM as the chain transfer agent via RAFT miniemulsion polymerization, leading to graft copolymer lignin-g-PAM-b-(MMA-*co*-BA). The thermal transition of these lignin-based hybrid acrylic resins was determined through a differential scanning calorimeter (DSC), and the thermal degradation of these hybrid resins was also examined using the thermogravimetric analysis (TGA) technique. In addition, the mechanical performance of the lignin-based MMA-*co*-BA hybrid resin was also studied with a stress–strain testing machine.

## 2. Materials and Methods

### 2.1. Materials

The lignin used in this study was the residue that resulted from the production of butanol using cornstalks, and it was supplied by Bairui Bio-Polyos Co. Ltd. (Songyuan, China). Acrylamide (AM, 99%) and potassium persulfate (KPS) were purchased from Beijing Biotopped Co. Ltd. and Aladdin Reagent Co. Ltd. (Shanghai, China), respectively. All other reagents, such as 2,2-azobisisobutyronitrile (AIBN), sodium dodecyl sulfonate (SDS), *N*,*N*-dimethylformamide (DMF), *n*-hexadecane (HD), butyl acrylate (BA), methyl methacrylate (MMA), ethyl acetate, and dichloromethane (DCM), were purchased from Sinopharm Chemical Reagent Co. Ltd (Shanghai, China).

### 2.2. Synthesis of the Lignin-Based Polymeric RAFT Agent

**Figure 1:**
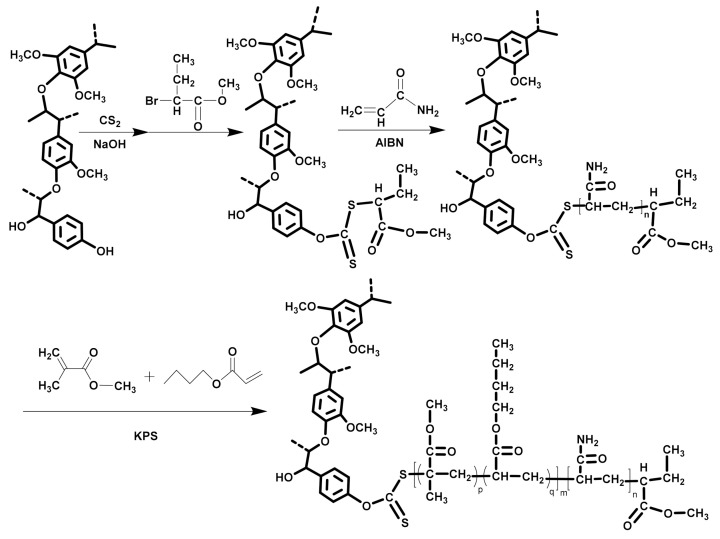
Schemes for the synthesis of lignin-graft-polyacrylamide (lignin-g-PAM) and methyl methacrylate copolymerized with butyl acrylate (lignin-based MMA-*co*-BA) hybrid resins.

Lignin xanthate was synthesized beforehand using a one-pot reaction, as illustrated in Figure 1. Lignin xanthate (1.0 g, 1.14 mmol xanthate group), AM (2.8 g, 38.00 mmol), and AIBN (62.4 mg, 0.38 mmol) were added to a 50 mL two-necked flask with a loading of 16 mL of DMF. Followed by purging with nitrogen (N_2_) for 45 min, the resulting solution was first heated to 70 °C under magnetic stirring and then maintained at the same reaction temperature for 24 h. The precipitate was separated using filtration after the mixture was cooled to ambient temperature. Finally, the obtained copolymer lignin-graft-polyacrylamide (lignin-g-PAM) was thoroughly purified using DCM and dried to a constant weight under a vacuum at 30 °C. The number average molecular weight gained in the experiment was about 6029 g/mol (molecular weight distribution = 1.54).

### 2.3. Synthesis of the Lignin-Based MMA-co-BA Hybrid Resins

Using lignin-g-PAM as the chain transfer agent, a typical reaction for the preparation of the lignin-based MMA-*co*-BA hybrid resin was carried out as follows: SDS (0.25 g) and KPS (0.09 g) were dissolved in 60 g of tap water and mixed with lignin-g-PAM (20 wt% of total MMA and BA weights); HD (0.1 g) and 5.6 g MMA were mixed with 4.4 g BA. Then, the mixture was treated using ultrasonic irradiation in a bath of ice water for 12 min. The resulting emulsion was immediately added to a 150 mL three-necked flask. After purging with N_2_ for 30 min, the system was heated to 75 °C and held constant for 6 h. Subsequently, the reaction gel was filtered out after the polymerization system was cooled down to ambient temperature. The monomer conversion of the above-mentioned RAFT miniemulsion polymerization was about 86% (Table 1). The extraction procedure using ethyl acetate was performed to remove impurities from the obtained block copolymers for analysis of the chemical structure and properties. The preparation scheme and the molecular model of the lignin-based MMA-*co*-BA hybrid resins are shown in Figure 1.

### 2.4. Analytics

#### 2.4.1. Attenuated Total Reflectance–Fourier Transform Infrared (ATR-FTIR)

The molecular structure of graft copolymers synthesized in the above-mentioned experiment was investigated using Fourier transform infrared (FTIR) spectrometry. The absorption spectra were recorded through a FTIR spectrometer (Nicolet iS10 Thermo Fisher Corporation, Madison, WI, USA) coupled with an attenuated total reflectance (ATR) accessory by scanning between 4000 and 500 cm^−1^. Transmission spectra data were collected by averaging 16 scans at a 4 cm^−1^ resolution.

#### 2.4.2. ^1^H Nuclear Magnetic Resonance (^1^H NMR)

The ^1^H NMR method was used to analyze the samples on an NMR apparatus (500 MHz, Bruker DRX500, Berlin, Germany). The spectra of the copolymers were obtained using tetramethylsilane as the internal reference at 25 °C with the relaxation time of 1.0 s; the employed solvent for the NMR test was dimethyl sulfoxide-d6 (DMSO-d6).

#### 2.4.3. Gel Permeation Chromatograph (GPC)

The average molecular weights of the copolymers were tested on a gel permeation chromatograph (Malvern Viscotek, Malvern, UK) with the refractive index detector at 40 °C. The efficient eluent taken in this test was DMF, flowing at a speed of 1.0 mL/min. According to the experimental data, the average molecular weights were calculated using a method in which the molecular weight curve was calibrated with polystyrene standards. The concentration of the samples in DMF, filtered using microfilters with a pore diameter of 0.22 μm, was around 3.0 mg/mL.

#### 2.4.4. Transmission Electron Microscopy (TEM)

Transmission electron microscopy (TEM; JEM-1000, Tokyo, Japan) was used to characterize the particle morphology and the size of the samples. In preparation for the TEM specimen, the graft copolymer and pure MMA-*co*-BA polymer solutions were diluted using deionized water and placed dropwise onto a copper grid, followed by drying at room temperature for TEM analysis.

#### 2.4.5. Differential Scanning Calorimetry (DSC)

The thermal behavior of the samples was investigated by the use of differential scanning calorimetry (Diamond DSC, Woodland, CA, USA). About 5 mg of each specimen was loaded and then tested with N_2_ flowing at a rate of 45 mL/min. To remove any thermal history, each specimen was scanned twice at a speed of 20 °C/min between −55 and 150 °C. Data collection was performed during the second scanning cycle. 

#### 2.4.6. Thermogravimetric Analysis (TGA)

The thermal decomposition of the lignin-based MMA-*co*-BA graft copolymers was studied via TGA (Netzsch STA409, Selb, Germany). For TGA analysis, a typical test was performed using around 10 mg of the specimen under N_2_ between 30 and 700 °C at a speed of 10 °C/min.

#### 2.4.7. Mechanical Performance

Test specimens were prepared by emulsion casting (thickness 0.2 mm) in an aluminum foil mold at 40 °C up to complete water evaporation. The dumbbell-shaped specimens were cut to 16.0 mm × 4.0 mm before testing at room temperature. Under the same conditions, three counterparts for each sample were measured. The stress–strain tests were conducted at a speed of 50 mm/min according to universal ISO 527-3.

## 3. Results and Discussion

### 3.1. Characterization of Lignin-g-PAM

The poor solubility of lignin xanthate in water retards its direct utilization in RAFT miniemulsion polymerization. As shown in Figure 1, the lignin-g-PAM macromolecular chain transfer agent was synthesized in the RAFT polymerization of acrylamide with lignin xanthate used as a chain transfer agent. After purification, functional xanthate groups were still attached in lignin-g-PAM.

**Figure 2:**
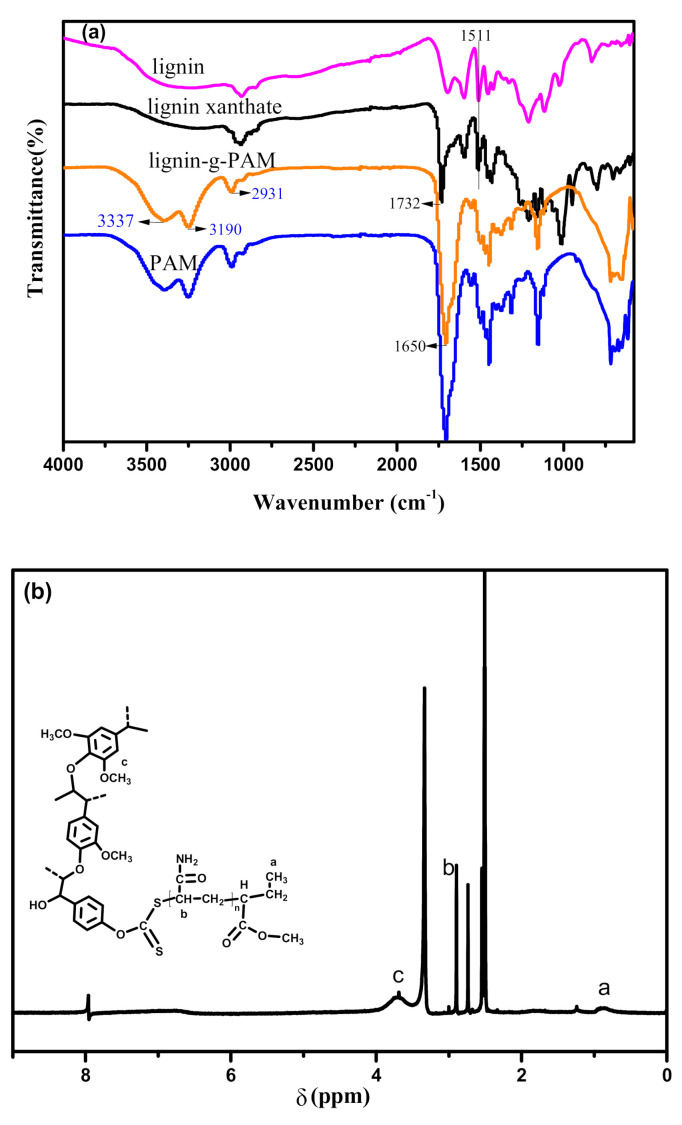
Attenuated total reflectance–Fourier transform (ATR-FTIR) spectra of lignin, lignin xanthate, lignin-g-PAM, and PAM (**a**), and ^1^H NMR spectrum of lignin-g-PAM (**b**).

As displayed in the ATR-FTIR spectra of lignin and lignin xanthate, the peak located at 1511 cm^−1^ is assigned to the aromatic ring from lignin [33]. However, this peak was not observed in the lignin-g-PAM spectrum due to the low concentration of the benzene ring. Compared with lignin xanthate, the ATR-FTIR spectrum (Figure 2a) of lignin-g-PAM displays a sharp peak at 1650 cm^−1^, which is associated with a carbonyl bond of the amide group [36]. In contrast, a wide absorption peak ranging from 3200 to 3590 cm^−1^, related to some –OH residue from lignin xanthate, was separated after a grafting reaction. Therefore, two absorption peaks at 3337 and 3190 cm^−1^, related to the N–H vibration and the N–H stretching of amine groups [36], also appeared. As presented in Figure 2a, the spectrum of lignin-g-PAM was very similar to that of PAM, because a considerable amount of PAM had grafted from lignin xanthate. Moreover, the polymerization of acrylamide with lignin xanthate also resulted in solubility changing, and, thus, lignin-g-PAM dissolved readily in water, while lignin xanthate had nearly no water solubility.

Two peaks appeared at 0.9 ppm and 3.7 ppm, as shown in the ^1^H NMR spectrum of lignin-g-PAM (Figure 2b), which are associated with the protons of the end –CH_3_ from the xanthates group and that of the methoxy group from lignin, respectively. 

### 3.2. RAFT Miniemulsion Polymerization and Properties of the Lignin-Based MMA-co-BA Hybrid Resin

To evaluate the efficacy of lignin-g-PAM in this article, RAFT miniemulsion polymerization of MMA and BA was investigated with different dosages of this macromolecular chain transfer agent. Then, we ran a group of polymerization reactions by simply varying the amount of lignin-g-PAM followed by changing the reaction temperature with the amount of lignin-g-PAM fixed at 40%. The resultant polymer was named lignin-g-PAM-b-(MMA-*co*-BA). The characteristics of the resulting latex prepared under various reaction conditions are summarized in Table 1. The gel fraction (GF) was determined as follows:

(1) $$\mathrm{GF}\left(\%\right)=W_{2}/W_{1}\times 100$$

In this equation, *W*_1_ is the total amount of the sample added to the lab flask, and *W*_2_ is the amount of that extracted sample corresponding to the residuals (gel fraction). 

As presented in Table 1, the RAFT polymerization reactions were carried out using acrylate monomers with lignin-g-PAM as the chain transfer agent at 75 °C to achieve high conversions above 87%, as determined by gravimetry. The polymerization rates were found to be much faster at a higher reaction temperature. Nearly 90% conversion was obtained at 85 °C after 6 h, while it took the same period of time for the same solution to reach as low as 63.3% conversion at 65 °C.

It was noted that the gel fraction was relatively low (0.02–0.11%) under all experimental conditions, and the polymerization of latex was stable throughout the entire process. It is also worth noting that these lignin-based MMA-*co*-BA hybrid latexes achieved high stability, of which the apparent viscosity and particle size did not display a minor increase over several months. The latexes resulting from our technique were stabilized by incorporating blocks of the hydrophilic monomer, acrylamide, in support of previous proposed findings [59]. Herein, lignin-g-PAM, a surface-active macromolecular RAFT agent composed of a hydrophilic polyacrylamide block and a hydrophobic lignin molecular chain, also had a favorable effect on latex stability. The relative stability of the dispersions is consistent with the results of the other systems reported earlier [60,61]. 

**Figure 3:**
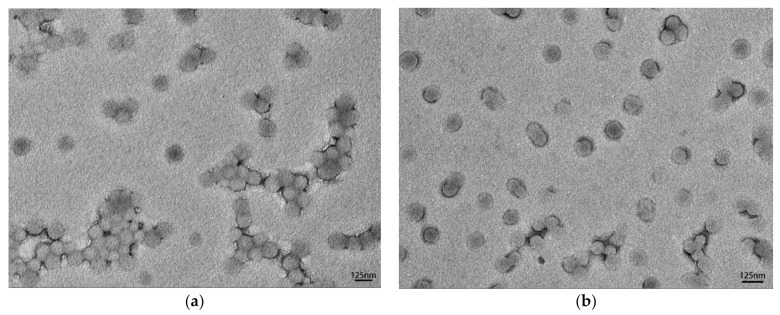
TEM images of the final latexes for lignin-based hybrid acrylate latex (**a**) and pure MMA-*co*-BA (**b**).

As a typical case, the morphology of the lignin-based MMA-*co*-BA hybrid latex synthesized using 40% lignin-g-PAM was examined by TEM in comparison to that of the pure MMA-*co*-BA. The TEM micrographs of the particle size of pure MMA-*co*-BA and 40% diluted lignin-g-PAM-b-(MMA-*co*-BA) latexes are shown in Figure 3a,b. As observed, the images of submicrometric particles are extremely similar for the two samples. The particle size of the latex prepared with 40% lignin-g-PAM (Figure 3a) was analyzed using ImageJ software and characterized by different particle diameters ranging approximately between 75 and 153 nm, with the majority around 95–125 nm. While the distribution of the particle size of pure MMA-*co*-BA latex existed between 62 and 151 nm, the vast majority was around 75–105 nm (Figure 3b). Furthermore, the mean particle size and standard deviation for 40% lignin-g-PAM-b-(MMA-*co*-BA) and pure MMA-*co*-BA were 108 ± 15 nm and 89 ± 12 nm, respectively. The particle size distribution in this study is very similar to the previous results obtained using RAFT miniemulsion polymerization [62]. These results outlined above lead us to believe that the RAFT miniemulsion polymerization was carried out successfully.

### 3.3. Characteristics of the Chemical Structure of the Lignin-Based Hybrid Acrylate Resins

**Figure 4:**
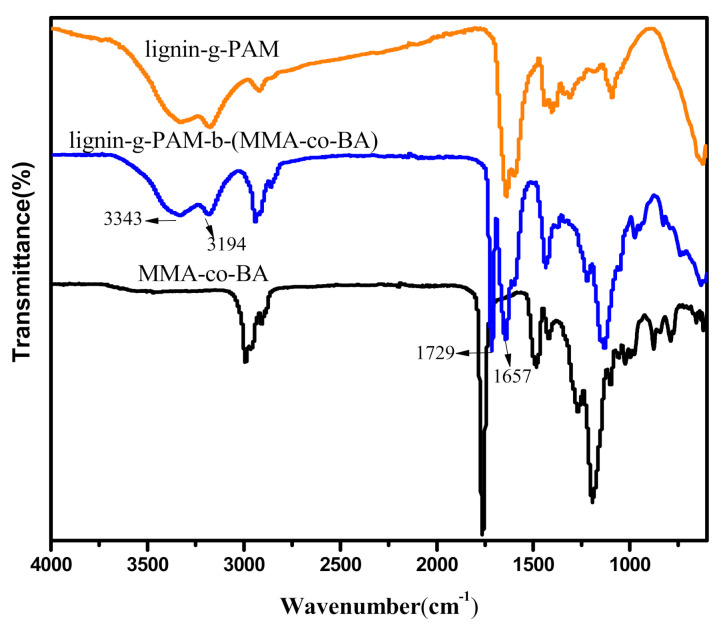
ATR-FTIR spectra of pure MMA-*co*-BA, lignin-grafted hybrid acrylate copolymer, and lignin-g-PAM.

The chemical structure of the lignin-based MMA-*co*-BA hybrid resin and the lignin-g-PAM chain transfer agent, compared to a reference MMA-*co*-BA copolymer, was analyzed using ATR-FTIR. Figure 4 shows the spectra of the above-mentioned copolymers.

**Figure 5:**
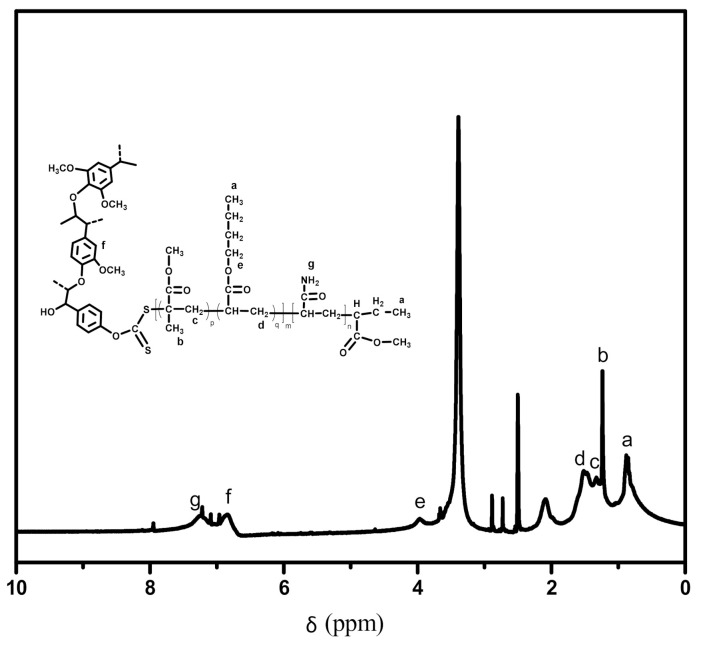
^1^H NMR spectrum of lignin-g-PAM-b-(MMA-*co*-BA).

The ATR-FTIR spectra of the lignin-based hybrid acrylic resin and lignin-g-PAM are extremely alike, but the vibration of the carbonyl group corresponding to 1729 cm^−1^ in the spectrum of the lignin-grafted MMA-*co*-BA resin confirms that the ester groups were incorporated into the lignin-g-PAM by RAFT miniemulsion polymerization. The strong absorption of peaks appeared at 3343 cm^−1^ and 3194 cm^−1^ in relation to the –NH_2_ stretching vibration of the unbonded amine group and that of the associated group, respectively. In addition, an absorption band at 1657 cm^−1^ associated with C=O amide was also found, similar to previous literature reports [36]. The ^1^H NMR spectrum of the lignin-based hybrid acrylic resin clearly showed a peak at 0.87 ppm mainly corresponding to the protons on the end –CH_3_ of the alkyl chain for BA (Figure 5).

### 3.4. Thermal Behavior

**Figure 6:**
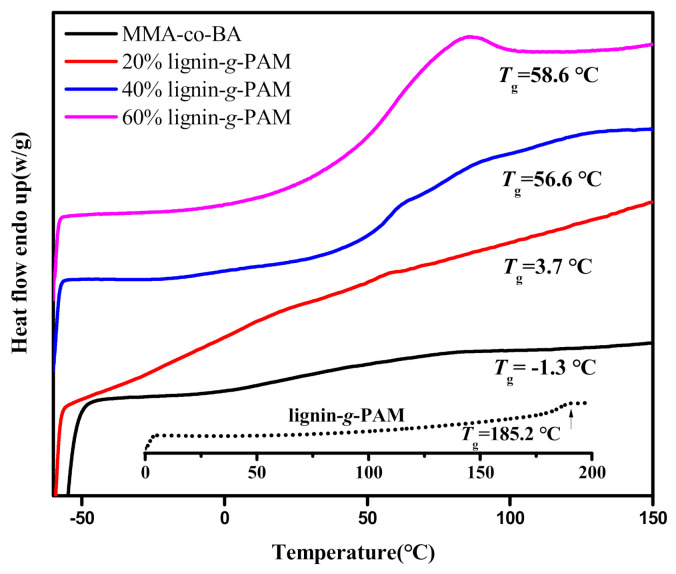
Differential scanning calorimetry (DSC) curves of lignin-g-PAM, pure MMA-*co*-BA, and lignin-grafted hybrid acrylic resins.

The glass transition temperature (*T*_g_), manifesting the macromolecular chain’s flexibility of the lignin-grafted MMA-*co*-BA copolymers and pure acrylic resin (MMA-*co*-BA), was characterized through DSC. Figure 6 displays the *T*_g_ values of the pure MMA-*co*-BA copolymer and lignin-grafted hybrid acrylic resins. As expected, in this experiment, the *T*_g_ of the lignin-grafted hybrid acrylic resins was higher than that of the linear acrylic copolymer, in which the MMA-to-BA ratio was equal by mole. With the mass ratio of lignin-g-PAM to acrylic monomers increasing, the *T*_g_ of the lignin-grafted hybrid acrylic resin increased. Thus, the higher *T*_g_ followed the higher percentage of the lignin-based macromolecular RAFT agent consistently. The higher *T*_g_ of the lignin-grafted hybrid acrylic resins could be attributed to the incorporation of lignin-g-PAM. As presented in Figure 6, the glass transition temperatures of the lignin-grafted MMA-*co*-BA copolymers (3.7, 56.6, and 58.6 °C) existed between that of lignin-g-PAM (185.2 °C) and pure MMA-*co*-BA (−1.3 °C), which demonstrates that lignin-g-PAM-b-(MMA-*co*-BA) block copolymers synthesized through the RAFT miniemulsion polymerization. The shift in *T*_g_ is due to forming amounts of the homogeneous mixed phase [63]. The results should further underline the successful block-*co*-polymerization contributing to the low gel fraction (0.02–0.11%) listed in Table 1.

**Figure 7:**
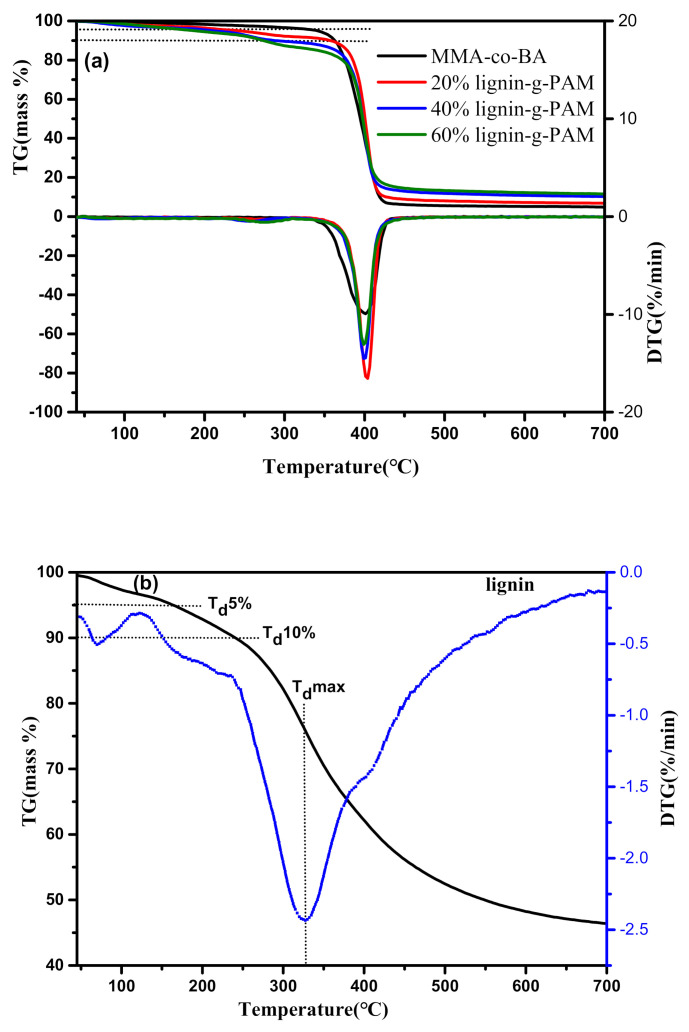
TG and DTG curves of the pure MMA-*co*-BA and lignin-based hybrid acrylic resins (**a**), and lignin (**b**).

The thermal stability of the pure acrylic resin and bio-based acrylic resins comprising lignin-g-PAM and lignin was investigated using TGA under an N_2_ atmosphere. Figure 7 shows these curves of TG and the resulting first derivatives (DTG, the rate of mass change as a function of temperature). The TGA results for T_d_ 5%, T_d_ 10%, T_d_ max, and carbon residue are summarized in Table 2.

The results show that the lignin-grafted MMA-*co*-BA resin started losing weight at lower temperatures in comparison to the pure acrylic copolymer. Furthermore, with the percentage of lignin-g-PAM increasing, the maximum decomposition temperatures of lignin-based hybrid acrylic resins decreased slightly from 410 to 390 °C. The above data demonstrate lower thermal stability of these hybrid acrylic resins than that of the linear MMA-*co*-BA copolymer, which could be mainly due to the microscopic phase separation resulting from the lignin with a lower molecular weight and, hence, a lower curing density of hybrid acrylic graft polymers [64]. 

As listed in Table 2, the T_d_ 5%, T_d_ 10%, and T_d_ max for lignin were lower than those of the MMA-*co*-BA and lignin-g-PAM-b-(MMA-*co*-BA) block copolymers. It was also observed that the T_d_ 5%, T_d_ 10%, and T_d_ max for pure MMA-*co*-BA were higher than those for all obtained lignin-g-PAM-b-(MMA-*co*-BA) block copolymers. The results partly stem from the lignin incorporated in these block copolymers.

In addition, the lignin used in this research consisted of hemicellulose, cellulose, and other components with lower molecular weight; the synthesized lignin-grafted hybrid acrylic resin could also possibly have contained some hemicellulose. Hemicellulose usually has poorer thermostability than lignin does; therefore, the lignin-based MMA-*co*-BA resins decomposed below a comparatively lower temperature (350 °C), partly because of the decomposition of hemicellulose incorporated into it [65]. Like other lignin-based polymers [64,65], these lignin-grafted hybrid acrylic resins decomposed with multiple complicated reactions occurring simultaneously. The above hybrid acrylic copolymers can also be depolymerized to CO_2_, CO, H_2_O, and monomers. The final carbon residue (CR) of the three lignin-based acrylic copolymers was 9.8–18.0% at 700 °C, higher than that of linear acrylic copolymer (CR 3.9%), which mainly resulted from lignin being incorporated into lignin-g-PAM-b-(MMA-*co*-BA) block copolymers.

### 3.5. Mechanical Property

Regarding the lignin-grafted MMA-*co*-BA hybrid resin, synthesized with 60% lignin-g-PAM, the resulting sample was very brittle at room temperature. The mechanical properties of the pure acrylic copolymer and lignin-grafted hybrid acrylic resins that were synthesized at two different percentages of lignin-g-PAM (20 and 40 wt%) were tested on a CMT4000 instrument.

**Figure 8:**
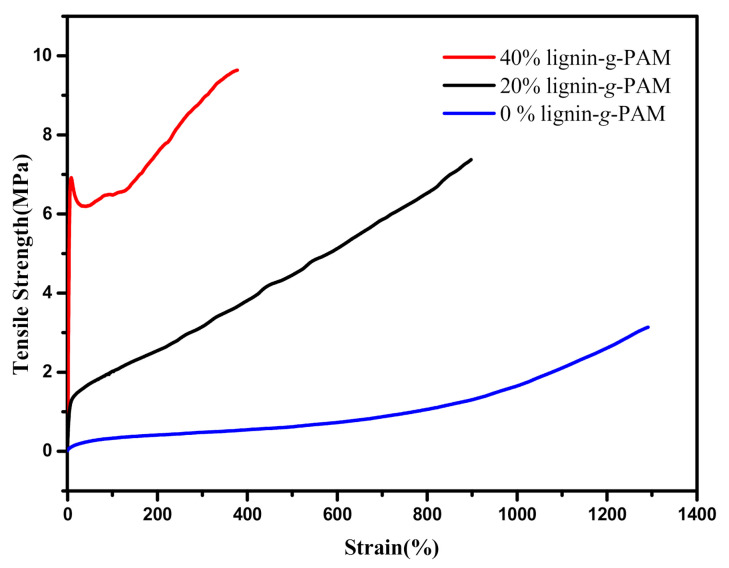
Strength–strain behaviors versus % of lignin-g-PAM for synthesis of lignin-grafted hybrid acrylic resins.

The tensile strength was obtained as the maximum applied stress at failure, and the Young’s modulus for the same samples was calculated from the slope of the stress–strain diagram. These recorded results are displayed in Table 3 and Figure 8. The data collected show that the mechanical properties (except failure strain, maximum load, Young’s modulus, and tensile strength) of the hybrid acrylate resins significantly increased as a higher percentage of lignin-g-PAM in lignin-based MMA-*co*-BA copolymers was used. As observed, a low failure strain in 40% lignin-g-PAM-b-(MMA-*co*-BA) may result in a poor deformation capacity and ductility of films at a higher ratio of the lignin-based macromolecular RAFT agent. One reason for this could be related to the more star-shaped architecture restricting chain mobility [25]. However, this hybrid acrylic resin prepared by RAFT polymerization using up to 40 wt% of lignin-g-PAM as the chain transfer agent showed a better Young’s modulus and tensile strength when compared to that of the linear MMA-*co*-BA. This suggests a great untapped potential for the manufacturing of tunable and environmentally friendly coatings or adhesives as a type of bio-renewable material to partially substitute fossil-based polymers.

## 4. Conclusions

The hybrid acrylic latexes incorporating a surface-active lignin-based macromolecular chain transfer agent were, without fail, prepared via RAFT miniemulsion polymerization. The ^1^ H NMR spectra, as well as the ATR-FTIR spectra, showed the attachment of acrylic molecular chains on the lignin. The *T*_g_ of lignin-based acrylic graft polymers was discovered to be higher than that of the pure acrylic copolymer in this experiment, of which the MMA-to-BA ratio was equal by mole. The TGA results revealed that the thermal stability of the acrylic hybrid resins was lower than that of the linear copolymer, which could have been caused by the microscopic phase separation of lignin-based acrylic graft polymers, partly due to the incorporation of lignin. However, these lignin-g-PAM-b-(MMA-*co*-BA) resins strongly demonstrated a better Young’s modulus and tensile strength as a result of the incorporated lignin when compared with the pure MMA-*co*-BA copolymer. The results obtained suggest that the lignin-based hybrid acrylic resins have good potential in the manufacturing of tunable bio-based materials as a partial substitute for petroleum-based copolymers.

## Figures and Tables

**Table 1:** Reversible addition–fragmentation transfer (RAFT) polymerization conditions and main characteristics of pure MMA-*co*-BA and lignin-based hybrid acrylate latexes.

Lignin-g-PAM Dosage (wt%)	Temperature (°C)	Conversion * (%)	Viscosity (mPa·s)	*M*_n_ (g/mol)	*M*_w_/*M*_n_	Gel Fraction (%)
0	75	85.0	34.6	129,000	3.70	0.03
20	75	86.1	36.6	638,000	2.84	0.02
40	75	88.3	36.3	802,000	2.55	0.05
60	75	87.5	28.3	446,000	2.25	0.11
40	65	63.3	26.3	237,000	2.39	0.03
40	85	89.3	26.5	1,007,000	4.17	0.08

* Measured by gravimetry.

**Table 2:** Thermogravimetric analysis results for T_d_ 5%, T_d_ 10%, T_d_ max, and carbon residue.

Sample	T_d_ 5% (°C)	T_d_ 10% (°C)	T_d_ max (°C)	Carbon Residue (%)
MMA-*co*-BA	341	362	410	3.9
20% lignin-g-PAM	242	358	403	9.8
40% lignin-g-PAM	210	288	393	16.3
60% lignin-g-PAM	188	273	390	18.0
lignin	163	240	327	44

**Table 3:** Tensile properties of pure acrylic copolymer and lignin-grafted hybrid acrylic resins.

Dose of Lignin-g-PAM (wt%)	Failure Strain (%)	Tensile Strength (MPa)	Maximum Load (MPa)	Young’s Modulus (MPa)
0	1216.50	2.60	2.36	0.27
20	863.96	6.56	8.90	17.03
40	419.62	8.53	24.01	198.78

## Data Availability

The data presented in this study are available upon request from the corresponding author.
